# Crowded developmental environment promotes adult sex-specific nutrient consumption in a polyphagous fly

**DOI:** 10.1186/s12983-019-0302-4

**Published:** 2019-02-18

**Authors:** Juliano Morimoto, Binh Nguyen, Hue Dinh, Anh The Than, Phillip W. Taylor, Fleur Ponton

**Affiliations:** 1 Department of Biological Sciences, Macquarie University, North Ryde, NSW 2109 Australia; 2 Department of Entomology, Vietnam National University of Agriculture, Hanoi, Vietnam

**Keywords:** Development, Larva, Nutrition, Predator-prey

## Abstract

**Background:**

The fitness of holometabolous insects depends largely on resources acquired at the larval stage. Larval density is an important factor modulating larval resource-acquisition, influencing adult survival, reproduction, and population maintenance. To date, however, our understanding of how larval crowding affects adult physiology and behaviour is limited, and little is known about how larval crowding affects adult non-reproductive ecological traits. Here, larval density in the rearing environment of the polyphagous fruit fly *Bactrocera tryoni* (‘Queensland fruit-fly’) was manipulated to generate crowded and uncrowded larval treatments. The effects of larval crowding on pupal weight, adult emergence, adult body weight, energetic reserves, fecundity, feeding patterns, flight ability, as well as adult predation risk were investigated.

**Results:**

Adults from the crowded larval treatment had lower adult emergence, body weight, energetic reserves, flight ability and fecundity compared to adults from the uncrowded larval treatment. Adults from the crowded larval treatment had greater total food consumption (i.e., consumption of yeast plus sucrose) relative to body weight for both sexes compared to adults from the uncrowded treatment. Furthermore, males from the crowded treatment consumed more yeast relative to their body weight than males from the uncrowded treatment, while females from the crowded treatment consumed more sucrose relative to their body weight than females from the uncrowded treatment. Importantly, an interaction between the relative consumptions of sucrose and yeast and sex revealed that the density of conspecifics in the developmental environment differentially affects feeding of adult males and females. We found no effect of larval treatment on adult predation probability. However, males were significantly more likely to be captured by ants than females.

**Conclusion:**

We show that larvae crowding can have important implications to ecological traits in a polyphagous fly, including traits such as adult energetic reserve, flight ability, and adult sex-specific nutrient intake. Our findings contextualise the effects of larval developmental conditions into a broad ecological framework, hence providing a better understanding of their significance to adult behaviour and fitness. Furthermore, the knowledge presented here can help us better understanding downstream density-dependent effects of mass rearing conditions of this species, with potential relevance to Sterile Insect Technique.

**Electronic supplementary material:**

The online version of this article (10.1186/s12983-019-0302-4) contains supplementary material, which is available to authorized users.

## Background

Resource acquisition during juvenile stages can have long-term implications through an animal’s life [1]. In holometabolous insects, in which larvae often share resources, larval density can be a key factor influencing access to nutrients needed for larval growth and development [2]. Larval density can significantly affect larval, pupal, adult and next-generation traits and, ultimately, the fate of groups and populations. For instance, when larvae are kept in crowded environments and/or poor nutritional conditions there may be a delay in pupation, an increase in mortality rate at the pupal stage, or reduced adult body size [3–16]. Because of the general trend in insects for body size to be positively associated with sexual performance (see e.g., [17–21]), crowding and poor larval nutrition tend to decrease adult sexual attractiveness and reproductive performance, including pre- and post-copulatory competitive ability (for males) and fecundity (for females) [10, 14, 15, 17, 18, 22]. Thus, larval crowding is an important ecological modulator of the strength of evolutionary forces such as sexual selection and sexual conflict [15].

Previous studies have largely focused on the effects of larval crowding on reproductive traits ([6, 23], e.g., sperm number and egg production [24]), while other aspects of adult physiology and ecology have been overlooked. As a result, very little is known about how larval crowing modulates traits that are not directly related to reproduction (here we referred to these traits as ‘ecological traits’). Key questions remain unanswered such as ‘how does larval crowding modulate the internal physiological and nutritional status of the organism in adulthood?’ and ‘can larval crowding affect the interactions between an individual and its environment (e.g., predators)?’ The answer to these questions will provide a better understanding of how larval crowding affects ecologically relevant factors, and will consequently help us gain a more complete perspective on the significance of larvae developmental conditions in broad ecological contexts.

Here, we investigated the influence of larval crowding on larval and adult ecological traits in the tephritid fruit fly *Bactrocera tryoni* (Froggatt) (Diptera: Tephritidae) (i.e., ‘Queensland fruit fly’). *Bactrocera tryoni* is one of the most destructive horticulture pest in Australia because of the wide variety of fruits that are used as hosts for oviposition [25]. Oviposition is influenced by multiple factors such as ovariole status, fruit size and quality, time of day as well as other ecological factors (e.g., temperature) [25–27]. Eggs are deposited in small batches of 4–20 eggs, which confers competitive advantage in exploring fruit patches in larvae natural environment [28]. Importantly, upon hatching, larvae are known to aggregate [29] with potential developmental benefits [30]. Furthermore, millions of *B. tryoni* are mass-reared for Sterile Insect Technique (SIT) whereby the density of larvae is a key variable in determining the quality of the adults and, consequently, the success of the released flies in nature [25]. Thus, better understanding *B. tryoni* behavioural implications of larval density not only advance our knowledge on life-history adaptations, but can also improve the management of this pest. We manipulated larval density to generate uncrowded and crowded larval treatments and used adult body weight as a proxy of the quality of the larval developmental environment. This approach has been widely used in insects (see e.g., [11, 15, 16, 23, 31–34]), including *B. tryoni* in which body size and weight of adult males are linked to mating probability and desiccation resistance [35–37]. We then tested whether larval density affected on (i) adult body energetic reserves, by measuring adult percentage of body lipid of adults; (ii) adult fecundity, by measuring the volume of eggs produced by adult females; (iii) individual feeding behaviour, by measuring the quantity of protein and carbohydrate, as well as total food intake of recently emerged adults; (vi) adult emergence and flight ability, by measuring the percentage of adults that emerged from pupae and the percentage of adults that were able to fly; and (v) probability of predation, by measuring the probability of predation by generalist ants (*Irydomyrmex spp.)*. In general, ants (including *Irydomyrmex spp* ants) are a common predator of invertebrates including tephritid fruit flies [38–40], and are likely to play an important ecological role on the survival *B. tryoni* in nature*.* More broadly, insights into how larval crowding affects adult fecundity, feeding behaviour, flight ability, and predation can reveal functional links between the effects of larval developmental conditions and physiological and behavioural factors that influence the fitness of individuals in adulthood. Based on the literature [3–18], we predicted (1) larvae from the crowded larval treatment to experience increased larval competition for nutrients, resulting in smaller and lighter adults, with significantly lower energetic reserves compared with the adults from the uncrowded larval treatment.

If prediction 1 is confirmed, then

1. Considering previous literature describing the positive association between female body size and egg productivity, including in *B. tryoni* [28], we predicted females from the crowded treatment to have lower egg production volume (used as a proxy for fecundity);
2. We predicted the physical constrains of smaller body sizes to result in lower absolute yeast, sucrose, and total food consumption for adults from the crowded larval treatment, although we predicted similar yeast, sucrose and total food consumption for adults of uncrowded and crowded larval treatments after controlling for adult body size (i.e., food consumption relative to body size);
3. We predicted the smaller body size and lower energetic reserves of adults from the crowded larval treatment to result in lower flight ability. This could affect the ability of adults to escape the attack, and we therefore hypothesised, after experiencing an ant infestation (see Methods), that adults from the crowded larval treatment would have higher predation risk. The understanding of how larval density influence adult flight ability allows us to predict the performance of mass-reared flies released into nature for Sterile Insect Technique, thereby advancing our understanding of the applied aspects of fruit fly biosecurity.

## Results

### Larval crowding results in adults with lower body weight, energetic reserves, and fecundity

**Fig. 1:**
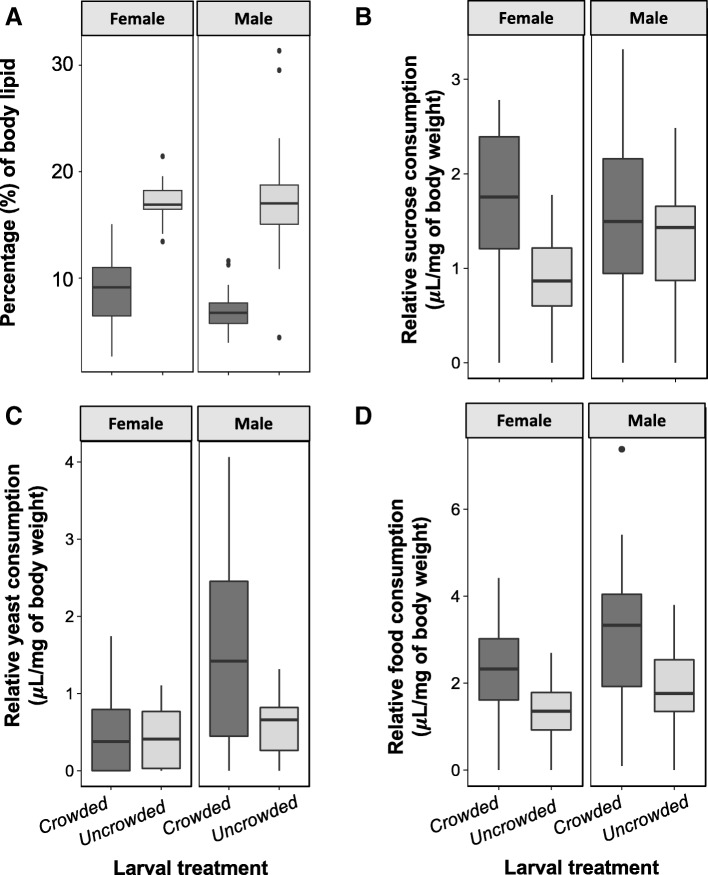
Effects of larval treatment on adult energetic reserves and relative food consumption. **a** Female and male energetic reserves (i.e., percentage of body lipid) across larval treatments. **b**-**d** Female and male relative sucrose (**b**), yeast (**c**) and total food consumptions (**d**) across larval treatments. Dark grey – Crowded treatment; Light grey – Uncrowded treatment

There was a significant effect of larval treatment on adult body weight, whereby adults from the crowded treatment had significantly lower body weight than adults from the uncrowded treatment (F_1,118_ = 980.508, *p* < 0.001, Additional file 1: Figure S1a, Additional file 2: Table S1). There was also a significant effect of sex on body weight with females having greater body weight than males (F_1,117_ = 30.646, *p* < 0.001, Additional file 2: Table S1), however there was no interaction between larval treatment and sex (Additional file 2: Table S1). Next, we quantified percentage of lipid stored relative to body mass using chloroform extraction and comparing adult body dry mass before and after lipid extraction (see Methods for details). Males and females from the crowded treatment had a significantly lower percentage of body lipid compared to males and females from the uncrowded treatment (*Males:* Kruskal-Wallis *χ*^2^ value = 22.548, *p* < 0.001, *Females:* Kruskal-Wallis *χ*^2^ value = 26.475, p < 0.001, Fig. 1a). Groups of adult flies from the crowded treatment had lower fecundity than groups of adult flies from uncrowded treatment (F_1,18_ = 30.111, *p* < 0.001, Additional file 1: Figure S1b).

### Feeding experiment: Sex-specific effects of larval crowding on yeast, sugar, and total food consumption relative to body weight

Females from the crowded treatment had significantly higher consumption of the sucrose solution relative to body weight compared to females from the uncrowded treatment (F_1,58_ = 23.917, *p* < 0.001, Fig. 1b), however there were no differences in the relative consumption of the yeast solution between females of either larval treatments (Fig. 1c, Additional file 2: Table S2). For males, however the consumption of the sucrose solution relative to their body weight was similar for the uncrowded and crowded treatments (Fig. 1b, Additional file 2: Table S2), our results revealed that males from the crowded treatment consumed significantly more of the yeast solution relative to body weight than males from the uncrowded treatment (F_1,54_ = 14.540, *p* < 0.001, Fig. 1c, Additional file 2: Table S2). There was also a weak but significant interaction between larval treatment and sex for the relative consumptions of the sucrose and yeast solutions (*Carbohydrate:* F_1,113_ = 4.391, *p* = 0.038, *Protein:* F_1,112_ = 9.289, *p* = 0.002, Additional file 2: Table S2). Males from the crowded treatment tended to consume more of the yeast solution relative to body weight than females from the crowded treatment, whereas males from the uncrowded treatment tended to consume more of the sucrose solution relative to their body weight than females from the uncrowded treatment (Additional file 2: Table S2, Fig. 1b-c). As a result, males and females from the crowded treatment consumed diets with a different balance of yeast-to-sucrose (Y:S) ratio (and consequently protein-to-carbohydrate ratio). While males from crowded environment consumed significantly higher Y:C ratios relative to their body weight compared with males from the uncrowded treatment (*Crowded Male Y:C ratio:* 2.2:1 ± 0.690; *Uncrowded Male Y:C ratio:* 1:1.5 ± 0.095; F_1,44_ = 5.453, *p* = 0.024), the opposite pattern was found for females, whereby females from crowded environment consumed significantly lower Y:C ratios relative to their body weight compared with females form the uncrowded treatment (*Crowded Female Y:C ratio:* 1:1.5 ± 0.053; *Uncrowded Female Y:C ratio:* 1:1.2 ± 0.065; F_1,39_ = 5.039, *p* = 0.030). There was also a significant interaction between larval treatment and sex for the relative Y:C ratio (F_1,83_ = 13.996, *p* < 0.001), whereby crowded larval treatment increased Y:C ratio balance of male feeding but decrease Y:C ratio balance of female feeding. Together, these results reveal a previously undescribed differential effect of larval density on the developmental environment on male and female nutrient consumption.

We then investigated total food consumption relative to body weight. Males and females from the crowded treatments consumed relatively more food than males and females from the uncrowded treatment (*Male:* F_1,54_ = 12.936, *p* < 0.001, *Female:* F_1,58_ = 16.781, p < 0.001, Fig. 1d). The interaction between sex and larval treatment was non-significant (F_1,112_ = 0.562, *p* > 0.4, Additional file 2: Table S2).

### Flight ability experiment: Larval crowding decreases adult emergence and flight ability

**Fig. 2:**
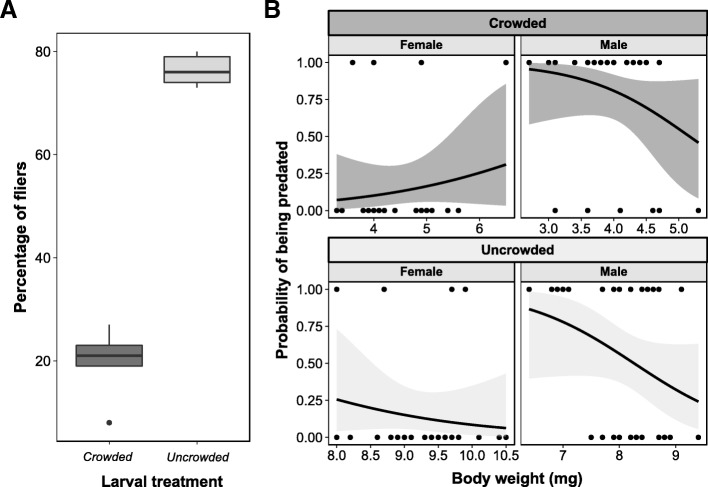
Effects of larval treatment on adult flight ability and probability of predation. **a** The percentage (%) of adult fliers across larval treatments. **b** Female and male probability of being predated by ants of the genus *Irydomyrmex* as a function of flies’ body weight (in mg) across larval treatments. Solid black curve represents the estimated probability based on a logistic regression. Shaded area around the solid curve represents the estimated standard deviation of the logistic regression model. Dark grey – Crowded treatment; Light grey – Uncrowded treatment

The percentages of non-emerged flies and partially emerged flies were significantly greater in the crowded treatment compared with the uncrowded treatment (Kruskal-Wallis; non-emerged flies: χ^2^ value = 6.050, *p* = 0.013; partially emerged flies: χ^2^ value = 6.859, *p* = 0.008; Additional file 2: Table S3). In addition, there was a striking effect of larval treatment on adult flight ability, whereby the percentage of fliers was significantly lower in the crowded treatment compared with the uncrowded treatment (Kruskal-Wallis *χ*^2^ value = 6.818, *p* = 0.009, Fig. 2a, Additional file 2: Table S3). There was no effect of larval treatment on the sex ratio of fliers (Kruskal-Wallis *χ*^2^ value = 1.097, *p* = 0.294).

### Predation: Body weight is a predictor of predation risk in males but not females, irrespective of the larval rearing experience

There was no statistically significant effect of larval treatment on predation risk of adults (*p* = 0.509, Additional file 2: Table S4). However, there was a significant interaction between adult body weight and sex (Residual Deviance = 114.41, *p* = 0.038, Additional file 2: Table S4), which was driven by males with lower body weight being more likely to be predated than males with higher body weight, irrespective of the larval treatment (Fig. 2b). Female predation risk was constantly low independently of body weight for both crowded and uncrowded treatments (Fig. 2b). There were statistically significant main effects of sex (Residual Deviance = 119.16, *p* < 0.001, Additional file 2: Table S4), and body weight (Residual Deviance = 153.84, *p* = 0.006, Additional file 2: Table S4) on the probability of flies being predated. There was no effect of the interaction between larval treatment and sex (Additional file 2: Table S4).

## Discussion

In this study, we demonstrate how larval density modulates adult body weight, energetic reserves, and fecundity, as well as male and female feeding behaviour, adult flight ability, and sex-specific risk of predation in *B. tryoni*. Our results confirmed our predictions 1 and 2 as well as previous findings that larval crowding results in lower adult body weight and fecundity [3–18, 22]. Moreover, as predicted, larval crowding also decreased adult body energetic reserves. Contrary to prediction 3, adults from the crowded treatment consumed significantly more food than adults from the uncrowded treatment relative to body weight. Food consumption was modulated differently between sexes though. Males from the crowded treatment consumed more protein than males from the uncrowded treatment relative to their body weight, whereas females from the crowded treatment consumed more carbohydrate than females from the uncrowded treatment relative to their body weight. The results also partly confirmed prediction 4 by revealing that larval crowding had a strong negative effect on adult emergence, and significantly decreased adult flight ability. Despite this, larval treatment did not affect adult probability of predation, although males of lower body weight in both treatments were significantly more likely to be predated by ants.

### Negative effects of larval developmental conditions on adult traits

Our results corroborate previous studies in other insect species showing that crowding during development negatively affect emergence by increasing the percentage of non-emerged and partially emerged adult flies, and results in adults with lower body weight, energetic reserves, and fecundity. Delays in pupation and/or increased mortality rate of the pupae caused by crowding have been shown in several species of Diptera (e.g., [5, 9, 16]), Lepidoptera [6, 13] and Coleoptera ([3, 7], although see [41] for the lack of an effect of larval density on adult body size in *Tenebrio molitor*). Moreover, crowding and poor nutrition during development has been shown to decrease adult body weight and fecundity in the yellow dung fly [8, 10], in *Drosophila melanogaster* [12, 14–16], in mosquitoes such as *Anopheles gambiae* [5] and *Aedes agypti* [11], and in the cowpea seed beetle *Callosobruchus maculatus* [4], whereas in Lepidoptera the relationship between larval rearing, nutrient limitation, and adult body weight is less clear (see for instance [6, 13, 42, 43]). Likewise, in the tephritid medfly *Ceratitis capitata*, protein deprivation in the larval stage decreased adult size for both males and females, delayed sexual maturity of males and egg productivity of females [44]. Here, our results suggest that larval rearing conditions could be a strong ecological modulator of *B. tryoni* performance in addition to the fitness costs incurred by lower fecundity*.* Crowding had a negative effect on the ability of adults to fly as previously observed in Lepidoptera (*Loxostege sticticalis*) [45]. A potential explanation for this effect is that larval crowding decreases the availability of protein for the developing larvae which decreases flight muscle development and flight ability [46]. This is similar to the patterns observed in aphids *Myzus persicae* in which both population density and diet play an important role on wing production [47, 48]. More studies on the physiological and anatomical effects of larval density and diet on flight muscle development are needed to confirm this hypothesis. Nonetheless, the lower flight ability of *B. tryoni* would likely affect the frequency of sexual encounters and significantly impair male courtship behaviour through impairments on pheromone release and acoustic signalling, which depends on wing vibration [49].

Larval crowding is likely to constrain access to key nutrients, especially protein that is required for larval development and growth [50, 51]. For instance, protein deprivation at the larval stage in Medly reduces adult emergence rate in ways that resemble our findings for crowding developmental environments, corroborating the similarities between crowded environments and poor nutrition at the larval stage in tephritid flies [52]. Adult *D. melanogaster* that experience protein deprivation as larvae also emerge with phenotypes similar to those of adults that develop in crowded larval rearing conditions, including small body size and weight, delayed development, and reduced fecundity [53]. In addition, genetic studies of *D. melanogaster* have provided important insights into the molecular pathways involved in the effects of protein deprivation on physiology. Mutations of the insulin-like receptor *InR* and the insulin-like receptor substrate *chico* generate adult flies with phenotypes similar to those adults that were either protein-deprived [53] or experienced crowded rearing conditions. Thus, it is likely that the larval crowding limits nutrient availability which might in turn affect growth pathways resulting in adults with lower body size and weight, potentially lower energetic reserves, and lower fecundity [54–56]*.* These studies of *D. melanogaster* provide a useful model for future studies in tephritids such as *B. tryoni*.

### Effects of larval developmental conditions on feeding

Our results showed that adults from the crowded larval treatment had not only similar absolute food consumption but higher food consumption relative to body weight. We also found a sex-specific nutrient consumption effect of larval treatment, whereby males from the crowded larval treatment had a greater consumption of the yeast solution (i.e., source of protein) relative to body weight than males from the uncrowded treatment. In general, tephritid fruit flies are anautogenous and rely largely on protein consumption as recently emerged adults (i.e., ‘post-teneral’) to complete sexual development [57–59]. Likewise, protein intake is an important nutritional factor modulating post-teneral development and sexual performance in *B. tryoni* (see e.g., [60], reviewed by [61]); and previous studies in other insect species have shown that adults from crowded conditions tend to grow bigger sexual organs relative to their body size, invest relatively more in mating, and reproduce relatively earlier than adults from uncrowded conditions [6, 10, 16, 23]. Therefore, it is possible that males from the crowded larval treatment alter their feeding in order to invest more in reproductive traits in ways that mitigate the costs of larval crowding on their fitness. Our results also showed that females from the crowded treatment consumed more of the sucrose solution (i.e., source of carbohydrate) relative to body weight than females from the uncrowded treatment; yet, the implications of this effect on reproduction are unknown. The higher yeast consumption relative to body weight of males from the crowded treatment could reflect an attempt to minimise delays in post-teneral development caused by poor developmental environment, whereby a higher yeast consumption could accelerate post-teneral development (reviewed by [61]). It is not clear why females from the crowded treatment did not exhibit the same pattern of increased yeast consumption relative to body weight, especially because females have greater need for protein than males [60, 62].

Our results not only showed a change in specific nutrient consumption, but also a shift in the consumption of protein-to-carbohydrate ratio (Y:C ratio). This is important because the dietary Y:C ratio plays a significant role in survival and sexual performance of male and female *B. tryoni* [63, 64]. In other insects, the dietary Y:C ratio has also been shown to affect immune response and lifespan, male fertility, as pre- and post-copulatory success, female reproductive rate, male mating effort, the growth of male secondary sexual traits and female fecundity [65–75] (see [76] for review). In our study, the Y:C ratio consumed by adults from crowded and uncrowded larval environments were protein-biased (in males) and carbohydrate-biased (in females), with potential downstream effects on lifespan and reproduction. Further studies are needed to investigate whether adults from crowded environments that are restricted on fixed Y:C ratios have different sexual performance than individuals allowed to select their own Y:C ratio.

### Sex-specific effects on the risk of predation

After the feeding experiment had ended, we experienced an ant infestation that attacked our experimental flies. Because flies were randomised and placed onto the same shelf (see Methods for details), this unplanned ant infestation allowed us to test whether larval crowding influenced interspecific predator-prey interactions. The data revealed that larval crowding did not affect predation risk of emerged adults. Surprisingly though, our results showed that within each larval treatment, males with lower body weight were more likely to be predated by ants than both males with higher body weight and females from all weights. It is possible that males are easier to catch than females. Alternatively, ants could have been attracted to males by volatiles that are produced by males and not females (or in lower quantities by females), either as a consequence of sex-specific difference in volatile profile (e.g., sex pheromone [77], different lipid profiles) [78–80]. However, *B. tryoni* males used in our study were immature and so are unlikely to have released pheromones [60, 81]. Interestingly, *Vespula germanica* wasps have evolved the ability to detect the odour emitted by male *Ceratitis capitata* ‘medflies’ in mating aggregations, which result in males being at higher predation risk than females [82]. In addition, the parasite *Psyttalia concolor –* which attacks larvae of the olive fly (*Bactrocera oleae*) –responds specifically to the male sex pheromone (*Z*)-9-tricosene as a guide to its foraging behaviour [83], suggesting that sex-specific volatile signalling can be exploited by parasites – and potentially by predators – in fruit flies. We recognise that our results should be interpreted with caution as our predation experiment was not fully controlled. This is nonetheless an exciting new avenue of research on the effects of the developmental environment on interspecific interaction and food webs.

## Conclusion

The majority of insect species have larval stages during development. Here we showed that crowding in the developmental environment of the larvae strongly modulate ecological, physiological and reproductive traits in a polyphagous fly. These findings provide a better understanding of the functional significance of developmental crowding to individuals when they reach adulthood, and highlight the importance of the developmental environment for the ecology and evolution of holometabolous insects. Our findings of the effects of larval density on adult traits can also assist the mass-rearing of flies for SIT, which largely relies on the optimum density of larvae for the production of high-quality flies, thereby contributing to the control of this important horticulture pest.

## Methods

### Fly stock and egg collection

Eggs were collected for 2 h from a laboratory-adapted stock of *B. tryoni* established in 2015 (> 20 generations old) that has been maintained in non-overlapping generations. Adults were provided a free-choice diet of hydrolysed yeast (MP Biomedicals Cat. n^o^ 02103304) and commercial refined cane sugar (CSR® White Sugar), while larvae were maintained using the diet developed by Chang CL, Vargas RI, Caceres C, Jang E and Cho IK [84] in a gel matrix [85] for the last 10 generations. All stocks and experiments were maintained in humidity (65 ± 5%) and temperature (25 ± 0.5 °C) controlled rooms with light cycles of 12 h light: 0.5 h dusk:11 h dark: 0.5 h dawn).

### Experimental design and statistical analyses

#### Larval rearing manipulation

To generate uncrowded and crowded larval treatments, 250 μL (ca. 3500 eggs) and 2 mL (ca. 28,000 eggs) of eggs were placed in clear plastic rearing trays containing 150 mL of the gel-based diet (*N* = 6 replicate trays *per* larval treatment), creating a density of ca. 23 eggs per gram of diet for the uncrowded treatment (known to generate high quality flies; see [85]) and ca. 187 eggs per gram of diet for the crowded treatment. After 7 d, the lids of the rearing trays were removed and the rearing trays were placed into larger plastic container with ca. 150 g of fine vermiculite for pupation. Pupae were sifted from the vermiculite three days after the rearing trays were placed onto the vermiculite and were placed in a 90 mm Petri dish inside a 5 L plastic container until adults emerged. Adults were randomly selected from a pool of adults from all trays, and provided water and a free-choice diet as described above for 12 h prior to our assessment of body weight, body lipid, and fecundity. All flies had unlimited access to water throughout the experiments.

#### Body weight

We sampled 30 freshly emerged (i.e., up to 24 h old) males and 30 freshly emerged females *per* treatment *(N total = 120*). The flies were killed by chilling and stored at − 20 °C for 24 h before weighing on a Sartorius® ME5 scale (0.0001 g precision). Data were normalized using a log transformation. We fitted a generalized linear model (GLM) with Gaussian distribution to test the effect of larval treatment, sex, and their interaction on adult body weight, followed by a Student-Newman-Keuls (SNK) posthoc test with significance level of 0.05. *P*-values were obtained from F-statistics.

#### Body lipid (energetic reserves)

We sampled 20 freshly emerged males and 20 freshly emerged females *per* larval treatment (*N total = 80)*, which were placed individually in 10 mL glass tubes, freeze-killed (− 20 °C) and dried at 60 °C for three days in a drying oven. Dried bodies were weighed on a Sartorius® ME5 scale (0.0001 g precision). 2 mL of chloroform (Sigma Aldrich®, Cat no. 288306) was then added to each tube which was then sealed with a rubber plug and held for 24 h before the chloroform was discarded. The chloroform procedure was repeated for three consecutive days to extract lipids [86]. Bodies were then left to dry at 60 °C for three days before we measured body weight after lipid extraction. The percentage of body lipid was calculated as the difference between the body weight before and after lipid extraction, standardized by the body weight of each fly before the lipid extraction multiplied by 100 (i.e., percentage of lipid relative to the body weight of each fly). Statistical inferences on the difference in the percentage of body lipid between larval treatments were made using non-parametric Kruskal-Wallis, as the data did not fulfil the assumptions of parametric models.

#### Fecundity

10 replicate groups *per* larval treatment (*N total =* 20) with 100 freshly emerged adult flies (equal sex-ratio) were assembled. Groups were maintained in 12.5 L Decor Tellfresh plastic cages (Cat no. 136000), in which an aperture (ca. 20 cm diameter) in the lid was made manually, and where a fine mesh with ca. 50 cm in length was attached with hot glue. This allowed us to handle flies inside the cage. Cages were kept sideways in the controlled conditions described previously. Egg collection started 10 d post-emergence, and was maintained for 10 d before flies were discarded. The total volume of eggs recovered from the oviposition bottles was estimated by volume, in which we allowed eggs to settle in a 1.5 mL Eppendorf tube, marked the volume of eggs with a permanent marker, and then used a 200 *μ*L pipette to deposit the same volume of water as of eggs (results in *μ*L). The number of dead females was also scored daily, and controlled for in our models by dividing the volume of eggs recovered by the number of females in the group (i.e., fecundity *per* female). We used ANOVA for statistical inference and transformed fecundity *per* female to fit the assumptions of the model (i.e., $\sqrt[4]{\mathrm{fecundity}\ \mathrm{per}\ \mathrm{female}}$). Plots are of the raw data.

#### Feeding experiment

We used a Capillary Feeder (CAFE) assay [87] adapted to *B. tryoni* (see [64]). Briefly, 30 recently emerged males and 30 freshly emerged females of each larval treatment (*N total* = 120) were weighed as described previously and housed individually in a transparent plastic cages (diameter = 4 cm, height = 6 cm), with access to two 30 *μ*L glass capillaries (Drummond Microcaps®, Cat no. 1–000-0300), each filled with either a 120 g/L solution of hydrolysed yeast solution as a source of protein (and micronutrients) or a 120 g/L solution of sucrose as a source of carbohydrates; flies also had ad libitum access to water. There were three apertures (ca 2 mm diameter) for air (see Supplementary Methods for details). We added 5 *μ*L of commercial red food dye (Brand: Pillar Box Red, Food Colouring, Queen®) that has no nutritional value into the sucrose solution (final dilution 1:10,000), allowing us to visualise the level of diet remaining in the capillaries. Evaporation of the diets from the capillaries was estimated using 8 control cages with no flies. Final consumption of yeast and sucrose solutions was performed using a digital calliper 24 h after the onset of the experiment to estimate the consumption of protein and carbohydrate, respectively. Diet consumption of the flies was corrected for evaporation in the control cages by subtracting diet consumption from the average evaporation of each diet in the control cages. Absolute food consumption was calculated as the sum of the consumption of the sucrose and yeast solutions over the 24 h of the experiment, whereas the relative food consumption was calculated as the absolute food consumption divided by the initial body weight of the fly. Y:S ratio was calculated as the ratio of the relative yeast and sucrose solution consumption. We fitted a GLM with Gaussian distribution for each sex to test the effect of larval treatment on male and female absolute and relative consumption of sucrose and yeast solutions, and relative Y:S consumption. To analyse the differences in sex-specific feeding patterns between larval treatments, we also fitted a GLM with Gaussian distribution in the full dataset, and included larval treatment, sex, and their interaction as predictors; the interaction term has information on whether larval crowding had a sex-specific rearing-dependent effect on nutrient consumption. We used a Student-Newman-Keuls (SNK) posthoc test with significance level of 0.05 to investigate the patterns of nutrient intakes. *P*-values were obtained from F-statistics. The analyses of the absolute carbohydrate, protein, and food consumptions are given as Supplementary Information (see Supplementary Methods, Supplementary Results and Additional file 1: Figure S2). One data point was an outlier with high leverage in the analyses of female protein consumption, and was excluded from the final analyses. The analysis that includes the high-leverage outlier is shown in supplementary information.

#### Flight ability experiment

Flight ability was performed following [85]. Briefly, 100 pupae of each larval treatment were placed in 90 mm Petri dish lids that were lined with black filter paper (*N* = 6 replicates *per* larval treatment). A 100 mm tall acrylic ‘flight tube’ (89 mm external diameter × 84 mm internal diameter) was placed onto the 90 mm Petri dish that contained the pupae (see Additional file 1: Figure S3 for schematic representation). The acrylic tube was painted black on the outside and coated with fine layer of unscented talcum powder on the inside to prevent flies from walking, instead of flying, out of the tube. Another 100 mm acrylic tube was selected although with no pupae. Both tubes were placed inside a mesh cage (32.5 cm × 32.5 cm × 32.5 cm, Megaview BugDorm-43030F) under a 20 W fluorescent tube at ca. 5 cm above the cages, which was on throughout the experiment. The empty tube allowed us to estimate adult flyback into the tube containing the pupae [85]. We measured the percentage of fully emerged, partially emerged flies and non-emerged flies. The percentage of fliers was calculated as the number of adult fliers (i.e., flies that were outside the tubes) + the number of adults in the flyback tubes + the same number as in the flyback tube in tube with the pupae (i.e., fliers that returned to the original tube), divided by the total number of pupae multiplied by 100 [88]. The sex ratio of the fliers was calculated as the number of male fliers divided by the number of female fliers. Because the flight ability data were non-normally distributed, and no transformation allowed us to reach normality, we used non-parametric Kruskal-Wallis tests to analyse the flight ability data. One outlier with high leverage in the analyses of non-emerged flies was excluded from the final analyses. The analysis that includes the high-leverage outlier is shown in supplementary information.

#### Predation observation

24 h after the onset of the feeding experiment (see above) and after feeding data had been recorded, we experienced an infestation of > 1000 ants of the genus *Irydomyrmex spp*. Flies of the feeding experiment were exposed to the ants for approximately 12 h (from dusk to dawn). As the feeding cages were placed randomly with regards to sex and treatment, and in the same location (a metal shelf at ca. 30 cm height from the floor), the ant infestation provided a natural experiment which allowed us to test whether larval crowding influenced the risk of flies being predated by the ants. Predictions were made before data analysis was conducted. We counted the number of flies that had parts of their bodies eaten by ants after the 12 h period during in which the flies were exposed to the ants. We then used a logistic regression (i.e., GLM with Binomial distribution) to investigate the relationship between the probability of flies being predated and fly body weight, larval treatment, sex, as well as the interactions between body weight and sex, and larval treatment and sex. *P*-values were obtained from the *χ*^2^ distribution.

## Additional files

Additional file 1: **Figure S1.** Effects of larval treatment on adult body weight and fecundity. **Figure S2.** Effects of larval treatment on absolute sucrose, yeast, and food consumptions. **Figure S3.** Schematic representation of the flight ability experiment**. (DOCX 392 kb)**

Additional file 2: **Table S1.** Complete analysis of the effect of larval treatment on adult body weight. Bold: *p* < 0.05. **Table S2.** Complete analysis of the effect of larval treatment on the sugar, yeast and total food consumption of males and females relative to their body weight. Bold: *p* < 0.001. Student-Newman-Keuls posthoc test. **Table S3.** Complete analysis of the effect of larval treatment on non-emergence, partial emergence, and the percentage of fliers (flight ability experiment). Bold: p < 0.001. **Table S4.** Complete analysis of the effect of larval treatment on fly predation risk. Bold: p < 0.001. **Table S5.** The qualitative effects of removing the outliers. **Table S6.** Complete analysis of the effect of larval treatment on the absolute sugar, yeast and total food consumption of males and females. Bold: p < 0.001. Student-Newman-Keuls posthoc test. (XLSX 44 kb)

## Abbreviations

°C: degrees Celsius
g: grams;
g/L: grams per Litre
h: hours
L: Litres
mg: milligrams
mL: millilitre
SIT: Sterile Insect Technique
Y:C: yeast-to-sucrose ratio in the diet
μL: microliter
