# Supplementary material for: Crowded developmental environment promotes adult sex-specific nutrient consumption in a polyphagous fly
Source: Front Zool. 2019 Feb 18;16:4. doi: 10.1186/s12983-019-0302-4 (PMC6379967; doi:10.1186/s12983-019-0302-4)
Supplement: Supplementary file 1 — Figure S1. Effects of larval treatment on adult body weight and fecundity. Figure S2. Effects of larval treatment on absolute sucrose, yeast, and food consumptions. Figure S3. Schematic representation of the flight ability experiment. (DOCX 392 kb) [file 12983_2019_302_MOESM1_ESM.docx]

**Supplementary Information: “Crowded developmental environment promotes adult sex-specific nutrient consumption in a polyphagous fly”**

Authors: Juliano Morimoto^1*^, Binh Nguyen^1^, Hue Dinh^1^, Anh The Than^1,2^, Phillip W. Taylor^1^, Fleur Ponton^1^

Author’s affiliations:

1 - Department of Biological Sciences, Macquarie University, NSW 2109, Australia

2 - Department of Entomology, Vietnam National University of Agriculture, Hanoi, Vietnam

*To whom correspondence should be addressed:

Juliano Morimoto

Address: Department of Biological Sciences, Macquarie University, NSW 2109, Australia

E-mail: juliano.morimoto@mq.edu.au

**Supplementary Results**

**Figure S1 – Effects of larval treatment on adult body weight and fecundity.** *(a)* Body weight (in mg) of males and females across larval treatments *(b)* Per female fecundity (in $\mu$L) of groups with adults from across larval treatments. Dark grey – Crowded treatment; Light grey – Uncrowded treatment.

***Absolute sucrose, yeast, and food consumptions***

Males from the crowded treatment consumed (in absolute terms) significantly less of the sucrose solution than males from the uncrowded treatment (F_1,55_ = 13.669, p < 0.001, Fig S2a, Table S6), although there were no differences in the absolute consumption of the yeast solution (Fig S2b, Table S6). Female from the crowded treatment consumed (in absolute terms) significantly less of the yeast solution than females from the uncrowded treatment, although there were no differences in the absolute consumption of the sucrose solution (F_1,57_ = 5.342, p = 0.024, Fig S2a-b, Table S6). There was a weak but significant interaction between larval treatment and sex for both the absolute consumptions of the sucrose and yeast solutions (*Carbohydrate:* F_1,113_ = 5.065, p = 0.026, *Protein:* F_1,112_ = 4.299, p = 0.040, Table S6) whereby in the crowded treatment, males tended to consume less of the sucrose solution than females whereas the opposite was found for the uncrowded treatment (Fig S2a). Furthermore, male consumed (in absolute terms) more of the yeast solution than females in the crowded treatment, but this pattern was far less evident in the uncrowded treatment (Fig S2b). Males and females from the crowded treatment had absolute food consumption similar to males and females from the uncrowded treatment (*Male:* F_1,54_ = 3.132, p > 0.08, *Female:* F_1,58_ = 2.189, p > 0.1, Fig S2c). The interaction between sex and larval treatment was non-significant (F_1,112_ = 0.144, p > 0.7, Table S6).

**Figure S2 – Effects of larval treatment on absolute sucrose, yeast, and food consumptions.** *(a-c)* Female and male sucrose *(a)*, yeast *(b)* and total food consumption *(c)* (in $\mu$L) across larval treatments. Dark grey – Crowded; Light grey – Uncrowded.

**Figure S3 – Schematic representation of the flight ability experiment.** Pupae were placed in a cage with two tubes (T1 ­= Tube 1; FB = flyback) and illuminated at the top see Methods for details).

**Supplementary Tables (given as separate Excel file)**

**Table S1 – Complete analysis of the effect of larval treatment on adult body weight. Bold:** p < 0.05

**Table S2 – Complete analysis of the effect of larval treatment on the sugar, yeast and total food consumption of males and females relative to their body weight. Bold:** p < 0.001. Student-Newman-Keuls posthoc test.

**Table S3 – Complete analysis of the effect of larval treatment on non-emergence, partial emergence, and the percentage of fliers (flight ability experiment). Bold:** p < 0.001.

**Table S4 – Complete analysis of the effect of larval treatment on fly predation risk. Bold:** p < 0.001.

**Table S5 – The qualitative effects of removing the outliers.**

**Table S6 – Complete analysis of the effect of larval treatment on the absolute sugar, yeast and total food consumption of males and females. Bold:** p < 0.001. Student-Newman-Keuls posthoc test.

**Supplementary References**

Chang, C.L., Vargas, R.I., Caceres, C., Jang, E. & Cho, I.K. (2006) **Development and assessment of a liquid larval diet for *Bactrocera dorsalis* (Diptera: Tephritidae).** *Annals of the Entomological Society of America,* **99,** 1191-1198.

Fanson, B.G., Weldon, C.W., Pérez-Staples, D., Simpson, S.J. & Taylor, P.W. (2009) **Nutrients, not caloric restriction, extend lifespan in Queensland fruit flies (*Bactrocera tryoni*).** *Aging Cell,* **8,** 514-523.

FAO/IAEA/USDA (2014) **Product quality control for sterile mass reared and released Tephritid fruit flies.** International Atomic Energy Agency Vienna, Austria.

Ja, W.W., Carvalho, G.B., Mak, E.M., de la Rosa, N.N., Fang, A.Y., Liong, J.C., Brummel, T. & Benzer, S. (2007) **Prandiology of Drosophila and the CAFE assay.** *Proceedings of the National Academy of Sciences,* **104,** 8253-8256.

Moadeli, T., Taylor, P.W. & Ponton, F. (2017) **High productivity gel diets for rearing of Queensland fruit fly, *Bactrocera tryoni*.** *Journal of Pest Science,* **2,** 507-520.

Ponton, F., Wilson, K., Holmes, A., Raubenheimer, D., Robinson, K.L. & Simpson, S.J. (2015) **Macronutrients mediate the functional relationship between Drosophila and Wolbachia.** *Proceedings of the Royal Society B-Biological Sciences,* **282,** 20142029.
